# Electrochemical DNA Sensor for Valrubicin Detection Based on Poly(Azure C) Films Deposited from Deep Eutectic Solvent

**DOI:** 10.3390/bios13100931

**Published:** 2023-10-18

**Authors:** Anna Porfireva, Ekaterina Begisheva, Vladimir Evtugyn, Gennady Evtugyn

**Affiliations:** 1 A.M. Butlerov’ Chemistry Institute, Kazan Federal University, 18 Kremlevskaya Street, 420008 Kazan, Russia; begischewa2000@gmail.com (E.B.); gennady.evtugyn@kpfu.ru (G.E.); 2 Interdisciplinary Center of Analytical Microscopy, Kazan Federal University, 18 Kremlevskaya Street, 420008 Kazan, Russia; vevtugyn@gmail.com; 3 Analytical Chemistry Department, Chemical Technology Institute, Ural Federal University, 19 Mira Street, 620002 Ekaterinburg, Russia

**Keywords:** deep eutectic solvent, electropolymerization, Azure C, electrochemical sensor, DNA sensor, valrubicin determination

## Abstract

A novel electrochemical DNA sensor was developed for the detection of the anthracycline drug, valrubicin, on the base of poly(Azure C) electropolymerized from the deep eutectic solvent reline and covered with adsorbed DNA from calf thymus. Biosensor assembling was performed by multiple scanning of the potential in one drop (100 µL) of the dye dissolved in reline and placed on the surface of a screen-printed carbon electrode. Stabilization of the coating was achieved by its polarization in the phosphate buffer. The electrochemical characteristics of the electron transfer were determined and compared with a similar coating obtained from phosphate buffer. The use of deep eutectic solvent made it possible to increase the monomer concentration and avoid using organic solvents on the stage of electrode modification. After the contact of the DNA sensor with valrubicin, two signals related to the intrinsic redox activity of the coating and the drug redox conversion were found on voltammogram. Their synchronous changes with the analyte concentration increased the reliability of the detection. In the square-wave mode, the DNA sensor made it possible to determine from 3 µM to 1 mM (limit of detection, 1 µM) in optimal conditions. The DNA sensor was successfully tested in the voltammetric determination of valrubicin in spiked artificial urine, Ringer-Locke solution mimicking plasma electrolytes and biological samples (urine and saliva) with a recovery of 90–110%. After further testing on clinical samples, it can find application in the pharmacokinetics studies and screening of new drugs’ interaction with DNA.

## 1. Introduction

Deep eutectic solvents (DES) present a new class of “eco-friendly” solvents due to their biocompatibility, non-toxicity and biodegradability [1,2]. DES could be used in many fields of analytical chemistry, e.g., extraction [3], chromatography [4], sorption and modification of various sorbents [5,6], and electrochemistry [7,8]. DES solvents consist of a donor and acceptor of hydrogen bonds. Their intermolecular interaction leads to the decrease in the DES melting point against those of initial components. Reline, a eutectic mixture of choline chloride and urea in a 1:2 molar ratio, is one of the most popular representatives of DES [9]. It is also widely used in various electrochemical applications, including energy storage [10], electropolymerization of various dyes [11], electrodeposition of metal nanoparticles [12] and their composites [13], etc. Performing electropolymerization in reline makes it possible to overcome a common drawback of the reaction in aqueous media, i.e., the low solubility of organic monomers. This improves the efficiency of electropolymerization together with the formation of new electropolymerized materials.

**Figure 1:**
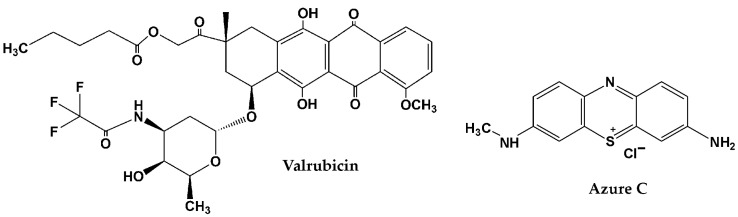
Chemical structures of valrubicin and Azure C (chloride salt).

Valrubicin (Figure 1), N-trifluoroacetyladriamycin-14-valerate, belongs to the family of anthracycline antibiotics that also includes doxorubicin, daunorubicin, idarubicin, epirubicin, nogalamycin and aclacinomycin [14,15]. All of these antineoplastic drugs are ordinarily used in cancer therapy. Excretion of anthracyclines and their metabolites happens mostly through biliary (60–80%) or urinary (40–20%) ways. As for anthracycline toxicity, suppression of bone marrow and gastrointestinal regeneration and cardiotoxicity should be mentioned [14]. Valrubicin is generally used as an intravesical agent for bladder cancer therapy [16,17]. Valrubicin demonstrates lipophilic properties and has low water solubility, so it rapidly enters the tumor cells through cell membranes [18,19]. As compared to doxorubicin, valrubicin causes less adverse events such as cardiac and skin toxicity, as far as it has minimal systemic absorption. Only 5% of patients suffered from systemic adverse events that were mild and elapsed a day later [19]. Low toxicity allows treatment with a higher concentration of valrubicin. Nowadays, only several examples of sensitive valrubicin determination are known by physicochemical methods: spectrofluorometry [20], spectrophotometry [21], high performance liquid chromatography (HPLC) with UV [22] and fluorimetric [23] detectors and electrochemical methods [24,25]. Electrochemical methods combine simple sample preparation, low cost, easy miniaturization, high sensitivity and rapidness which are highly demanded in point-of-care testing (POCT) devices. To date, only two examples of electrochemical valrubicin sensor were developed [24,25]. The integration of the carboxylated multiwalled carbon nanotubes, CeO_2_, Au nanoparticles and functionalized glassy carbon microspheres provided valrubicin detection with the linear range from 71 to 580 nM and a limit of detection (LOD) of 1.56 nM [24]. The Au electrode modified with multiwalled carbon nanotubes, ethylene diamine and Au nanoparticles made it possible to record the valrubicin reduction peak linearly, depending on its concentration in the range from 0.5 to 80 µM (LOD of 0.018 µM). To date, no works have been devoted to biosensor-based determination of valrubicin.

Azure C (N-methylthionine, see chemical structure in Figure 1) can be electrochemically converted into the polymer that is deposited on the electrode surface and applied to the assembly of electrochemical sensors, biofuel cells, supercapacitors and photovoltaic devices [26,27,28,29,30,31]. Like other polymeric forms of phenothiazine dyes, poly(Azure C) (PAC) possesses reversible redox signals attributed to the phenothiazine core and good stability at physiological pH [32].

DNA as a biological target of valrubicin can play the role of a specific bioreceptor accumulating the drug molecules due to their planar structure and ability to interact with double-stranded DNA molecules [33]. Such an interaction results in classical or threading intercalation depending on the intercalator structure [34]. If DNA molecules are adsorbed on the PAC layer, intercalation and electrostatic interactions will affect the redox equilibrium of the PAC underlying film and hence will be detectable using the voltammetry technique.

In this work, the determination of valrubicin with the DNA sensor was reported for the first time. The proposed DNA sensor involved the PAC layer deposited from reline required for improving the characteristics of electropolymerization and exclusion of the necessity in organic solvents. Then, calf thymus DNA was physically adsorbed on the polymer layer via electrostatic interactions. After the contact with the valrubicin sample, two signals attributed to both the drug and PAC were observed. Monitoring their simultaneous changes with valrubicin concentration increases the durability of the DNA sensor. Fast and reliable valrubicin measurements with the developed DNA sensor are demanded in medicine and pharmacology for pharmacokinetics of the anticancer drug, personal dose correction and the screening of new drugs interacting with DNA.

## 2. Materials and Methods

### 2.1. Reagents

Azure C chloride was purchased from Alfa Aesar (Ward Hill, MA, USA), choline chloride from Acros Organics (Gell, Belgium), deoxyribonucleic acid (DNA) from calf thymus, and valrubicin and urea from Sigma-Aldrich (Darmstadt, Germany). Other reagents were of analytical grade. All the reagents were used without further purification. Deionized Millipore Q^®^ water (Simplicity^®^ water purification system, Merck Millipore, Mosheim, France) was used for the preparation of working solutions. Phosphate buffer (PB) prepared from 0.1 M NaH_2_PO_4_ and 0.1 M NaNO_3,_ followed by pH adjustment, was used in voltammetric investigations including pH dependence establishment in the pH range from 2.0 to 10.0.

### 2.2. Apparatus

Voltammetric measurements were performed at ambient temperature using bipotentiostat-galvanostat μStat 400 Metrohm DropSens (DropSens, S.L., Asturias Llanera, Spain). Screen-printed carbon electrodes (SPCEs) were manufactured with the DEC 248 printer (DEK, London, England). Lomond PE DS Laser Film (thickness 125 μm, Lomond Trading Ltd., Douglas, Isle of Man) was used as a support. PSP-2 silver paste (Delta-Paste, Moscow, Russia), carbon/graphite paste C2030519P4 (Gwent group, Pontypool, UK) and dielectric paste D2140114D5 (Gwent group) were applied for printing conducting tracks, carbon pads and insulating layers, respectively. The layers were hardened at 80 °C. The electrode group had dimensions of 11 mm × 27 mm, with the geometric working area of the working electrode equal to 3.8 mm^2^. All the potentials were given against the pseudo-reference electrode Ag/AgCl printed together with other electrodes. The SPCEs were connected with boxed connectors (DSC, Metrohm) for screen-printed electrodes. Cyclic voltammetry (CV) was used for electrochemical characterization of the PAC layers deposited either from aqueous media (PAC1) or reline (PAC2 and PAC3). CV and square-wave voltammetry (SWV) were used for the valrubicin determination.

Scanning electron microscopy (SEM) images of Metrohm DropSens DRP-110 SPCEs (DropSens, S.L., Asturias Llanera, Spain), covered with modifying coatings, were obtained with a Merlin™ (Carl Zeiss AG, Oberkochen, Germany) high-resolution field emission scanning electron microscope equipped with an energy dispersion spectrometer AZtec X-Max.3. ZeissSmartSEM software (version 6.06) was used for image treatment.

Statistical data analysis and graphs presentation were performed with OriginPro 8.1 software (OriginLab Corp., Northampton, MA, USA).

### 2.3. Poly(Azure C) Electropolymerization and DNA Sensor Assembling

DES reline was prepared by mixing 0.1396 g of choline chloride and 0.12 g of urea (molar ratio 1:2). To prepare 0.1 M Azure C solution, 5.6 mg of the dye was added to the reline composition described above. The mixture was homogenized with vortex for 1 min and then sonicated for 30 min. This protocol was chosen because ultrasound-assisted synthesis was earlier found to be fast and efficient for reline synthesis [35]. After that, 100 µL of reline containing 0.1 M Azure C was drop-casted on the surface of the working electrode and the potential was cycled (20 cycles between −1.2 and 1.2 V for the PAC2 layer or between −0.7 and 1.2 V for the PAC3 layer; scan rate of 0.15 V/s). Then, the electrode was stabilized by performing ten consecutive scans in a fresh drop of 0.1 M PB, pH = 7.0 in the potential range from −0.65 to 0.50 V and scan rate 0.15 V/s. Thereafter, a voltammogram was recorded in above mentioned conditions to record the redox peaks of the PAC2 (PAC3) layer. DNA from calf thymus was immobilized on the electropolymerized PAC2 by physical adsorption. For this purpose, 2 µL of 1 mg/mL DNA solution were drop-casted on the working electrode surface and capped with plastic tubing for 20 min to prevent drying the solution. Finally, the electrode was washed to remove unbonded DNA molecules. All the procedures were performed at ambient temperature.

The Azure C concentration chosen for the electropolymerization from aqueous media was equal to 0.2 mM because of the lower solubility of the dye against reline. The SPCE was covered with 100 µL of 0.2 mM Azure C dissolved in 0.1 M PB (pH 7.0), and the potential of the electrode was cycled 20 times between −0.7 and 1.2 V (scan rate 0.15 V/s). The resulting PAC1 layer was stabilized by ten consecutive scans of the potential performed in a fresh drop of 0.1 M PB (pH 7.0) in the potential range from −0.7 to 0.5 V (scan rate 0.15 V/s). Then, a voltammogram was recorded in the potential range from −0.7 to 0.5 V (scan rate 0.15 V/s) to record the redox peaks of the PAC1.

### 2.4. Valrubicin Measurements, Real Sample Analysis

The surface of the PAC2/DNA-modified electrode was covered with 5 µL of the valrubicin solution (3 µM–1 mM). First, the stock solution of 0.01 M valrubicin in ethanol was diluted with 0.1 M PB containing 0.1 M NaNO_3_ (pH 7.0) immediately before the incubation. The electrode set was covered with the plastic tube for 20 min to prevent drying, then washed with deionized water and air-dried at ambient temperature. After that, the SPCE was covered with 100 µL of 0.1 M PB containing 0.1 M NaNO_3_ (pH 7.0), and CV or SWV were recorded. CV were registered in the same PB solution in the range from −0.65 and 0.50 V at the scan rate of 150 mV/s. SWV voltammograms were recorded in the same media by the potential scanning between 0.55 and −0.60 V, with accumulation time 5 s at 0.55 V, potential step 0.005 V, amplitude 0.002 V and frequency 20 Hz.

Ringer-Locke solution was taken to mimic plasma electrolytes (9 g/L NaCl, 0.42 g/L KCl, 0.5 g/L NaH_2_PO_4_·2H_2_O, 0.32 g/L CaCl_2_·2H_2_O, 0.1 g/L NaHCO_3_, 0.3 g/L MgSO_4_ and 1.5 g/L D-glucose) with pH 7.0.

Artificial urine samples contained 20 mM KCl, 49 mM NaCl, 15 mM KH_2_PO_4_, 10 mM CaCl_2_, 18 mM NH_4_Cl and 18 mM urea. Human urine and saliva samples were collected from conditionally healthy volunteers. The pH value of artificial and human urine samples was corrected until it reached 7.0. Urine samples were filtrated through the filtration paper if sediment appeared at the pH correction. Saliva samples were centrifugated at 10 000 rpm for 10 min and then used with no pH correction.

## 3. Results and Discussion

### 3.1. Azure C Electropolymerization on Screen-Printed Carbon Electrodes from Aqueous Solution

**Figure 2:**
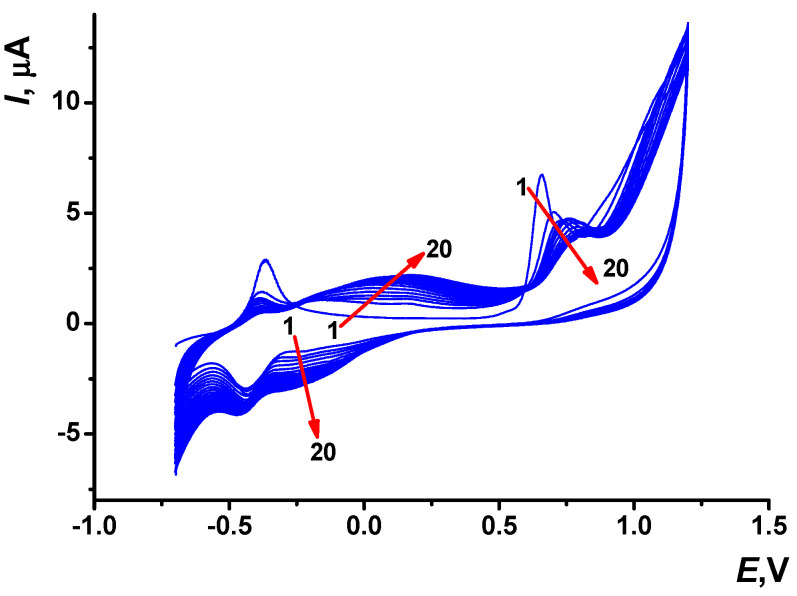
Multiple cyclic voltammograms recorded on the SPCE in 0.1 M PB containing 0.2 mM Azure C, pH 7.0, scan rate 0.15 V/s and 20 cycles. Arrows indicate changes with increased number of cycles.

Multiple cycling of the potential of 0.2 mM Azure C in PB (see conditions in the Section 2.3) was performed, and specific changes of the peaks on voltammograms were observed (Figure 2).

At the first scan, only one pair of the monomer redox peaks was found in the cathodic area at about −0.37 and −0.43 V. In the anodic area, the irreversible oxidation wave at about +0.66 V corresponded to the cation radical formation initiating electropolymerization. Low potential of the cation radical formation is rather typical for phenothiazine derivatives containing the primary amino group [36]. The signal at +0.66 V was shifted to more anodic potentials and became lower with the increased number of the potential cycles. From the second potential cycle, a pair of redox peaks attributed to the polymeric form of Azure C appeared at the potentials, more positive than those of the monomeric form. The appropriated peak potentials reached +0.17 and −0.30 V up to twentieth cycle. The peaks attributed to the electropolymerization products broadened with continued cycling of the potential.

Then, the electrode modified with PAC1 was covered with a fresh drop (100 µL) of the PB and electrochemically stabilized (see description in Section 2.3). Two pairs of the redox peaks related to the monomeric and polymeric forms of Azure C were observed on the voltammograms. Such a behavior was typical to the Azure group of phenothiazine dyes (Figure S1a, Electronic Supplementary Information) [37]. During the stabilization, peak currents of the Azure C monomer decreased from the first to tenth cycle probably due to leaching out the entrapped monomer from the surface layer. The currents attributed to the polymeric form were more stable and changed slightly in the PB buffer. Both pairs of peaks were then considered for the assessment of the PAC1 electrochemical parameters.

**Figure 3:**
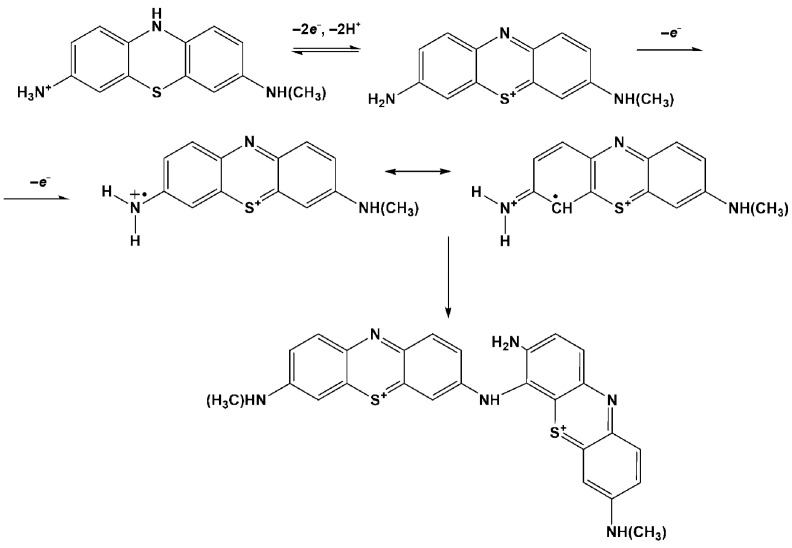
Tentative mechanism of Azure C electropolymerization.

The mechanism of electropolymerization assumes the formation of cation radical or dication [38,39] that is then bonded to the phenothiazine core via the “head-to-tail” (Figure 3) or “ring-to-ring” coupling mechanism [40,41]. In both cases, the phenothiazine core of the product preserves the ability of reversible oxidation–reduction, similarly to that of the monomeric dye. For this reason, the following interaction of the electropolymerization product with negatively charged DNA seems to be the same, irrespective of the detailed mechanism of electropolymerization, which remained the subject of the following investigations. The tentative mechanism of the reaction is presented in Figure 3. The first line corresponds to the pair of redox peaks observed at about −0.5 V on voltammograms and the second one to the initiation of polymerization.

As the PAC layer is present in the oxidized (positively charged) form, anionic counter ions can be implemented in the film. Commonly, they are taken from the supporting electrolyte present in the solution in electrochemical measurements, but after DNA adsorption, they are mostly substituted with the negatively charged DNA molecules. Such a process simplifies the formation of the PAC/DNA composite film and promotes the following determination of valrubicin as the DNA intercalator.

Regarding the thickness of the deposited PAC layer, the assessment is complicated by uncertainty in the stoichiometry of electropolymerization reaction and the rather high roughness of the underlying surface, preventing the use of electron microscopy. Similar studies on electropolymerization in potentiodynamic electrolysis reported on 10–100 nm thick films deposited [42,43]. DNA implementation via physical adsorption based on electrostatic interactions results in the deposition of a monolayer or a 2 nm increase in the film thickness.

### 3.2. Azure C Electropolymerization on Screen-Printed Carbon Electrodes from Reline

Monomer solubility and voltammetric curve morphology observed during the electropolymerization in reline differed from those observed in aqueous media. D’Agostino et al. [44] showed that the diffusion in DES was rather similar to that in ionic liquids and could be described with the hopping mechanism rather than with the conventional random walk approach. At 25 °C, the reline density is about 1.25 g/mL, with viscosity of η = 750 mPa·s. To reach a high Azure C redox signal in reline, the dye concentration was elevated to 0.1 M. Two different potential ranges were tested for electropolymerization, a wide one between −1.2 and 1.2 V and a narrow one between −0.7 and 1.2 V (scan rate 0.15 V/s). Despite the fact that in the wide potential range, the oxygen reduction reaction participated in the electrochemical process, the choice of the narrow range of potentials resulted in less efficiency of electropolymerization.

**Figure 4:**
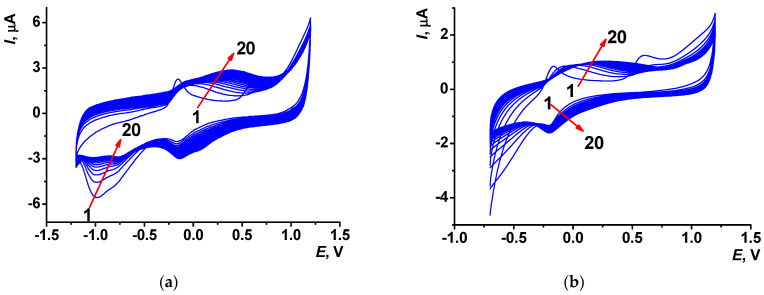
Multiple cyclic voltammograms recorded on the SPCE in reline with 0.1 M Azure C; (**a**) between −1.2 and 1.2 V and (**b**) between −0.7 and 1.2 V, scan rate 0.15 V/s, 20 cycles. Arrows indicate changes with increased cycle number.

Multiple cycling voltammograms recorded in reline containing 0.1 M Azure C are presented in Figure 4. It is worth noting that the shift of the peak attributed to the Azure C cation radical formation to the cathodic area (+0.61 V in reline against +0.66 V in PB) indicates facilitation of the electron transfer. The monomer redox peaks appeared at −0.16 and −0.19 V, whereas polymer redox peaks potentials depended on the electropolymerization regime. In the wide range electropolymerization potentials, they shifted to +0.39 and +0.10 V at the 20th cycle; for the narrow range of potential scanning, cathodic signals of the polymer were poorly resolved and the anodic potential was equal to +0.26 V. The layers electropolymerized from reline were marked as PAC2 and PAC3. They exerted higher stability against PAC1 (Figure S1b,c).

Electropolymerization in a wide potential range performed in the presence of dissolved oxygen can be affected by reactive oxygen species formed at these potentials. They can be implemented in the electron transfer resulting in the polymer chain growth together with the Azure C cation radical. As a result, peak currents recorded for the PAC2 coating were twice as high as those of PAC3. Electrochemical parameters of electron transfer were assessed for PAC2 and PAC3 modifier layers.

### 3.3. Comparison of Electrochemical Properties of Poly(Azure C) from Reline and Phosphate Buffer

For all of the types of the synthesized coatings, the dependence of the peak potentials on the pH and scan rate were compared and appropriate electrochemical characteristics assessed. Ten cycles of the potential were performed in the 100 µL of PB to stabilize the signals before peak characteristics measurements. In the case of PAC2, redox peaks of monomeric form were poorly resolved after the stabilization procedure, so only polymeric currents were processed.

The pH variation affected the signals of monomeric and polymeric forms of PAC1, PAC2 and PAC3 in a different manner. Thus, the PAC1 redox peak currents of the monomer showed a local maximum at pH = 5. The oxidation peak currents of polymeric form barely changed, with pH slightly increasing in neutral and weakly alkaline media. The reduction peak currents of the polymeric form increased linearly with the pH in the range from 2.0 to 8.0. For the PAC2 oxidation peak currents of polymeric form, a clearly defined maximum at pH = 5.0 was observed. The reduction peak currents of polymeric form were stable in the weak acidic and neutral media. They slowly decreased in strong acidic solutions and increased in alkaline solutions. In the case of PAC3, the oxidation peak current of monomeric form was stable in the weak acidic and neutral media, arising at pH = 2.0. The reduction peak current attributed to the monomer was about constant in the whole pH range considered. The redox peaks of the PAC3 polymeric form were smaller but changed similarly to the redox peaks of the PAC2 polymeric form (Figure S2).

The half-sum of the peak potentials of the monomeric and polymeric forms of the PAC was considered as an equilibrium redox potential and denoted as *E_m_*. For the PAC1 coating, the monomer signal slope of the *E_m_*–pH dependence exceeded the Nernstian slope in the acidic medium (85 mV/pH, pH = 2.0–4.0). It could be related to the unsteady conditions of the H+ transfer in the surface layer. In addition, it was supposed in the literature that three electrons and two hydrogen ions could be transferred to the poly(Neutral red) coating in acidic conditions (pH = 0.5–4.3) with a super-Nernstian slope of the dependency [45]. Similar to this explanation, a protonated form of Azure C can be involved in such equilibria. In the pH range from 4.0 to 8.0, the monomeric form demonstrated a slope of 47 mV/pH that formally corresponded to the transfer of three electrons and two hydrogen ions. The own buffer capacity of the PAC1 layer also contributes to the deviation from theoretical Nernstian slopes of the pH dependencies. The PAC1 polymeric form showed the slope close to the Nernstian value of 64 mV/pH in the pH range from 2.0 to 5.0. Thus, an equal number of electrons and hydrogen ions were transferred in the limiting step of the electrode reaction. When the pH ranged from 5.0 to 8.0, the slope of 24 mV/pH corresponded to the transfer of a single hydrogen ion and two electrons.

For the PAC2 coating, the slope of the *E_m_*–pH dependence of polymeric form was equal to 39 mV/pH in acidic and neutral media (pH = 2.0–7.0) and to 54 mV/pH in the alkaline solution (pH = 7.0–10.0).

For PAC3, appropriate dependence of monomeric form showed the slope of 80 mV/pH in the range of pH from 2.0 to 4.0 and 42 mV/pH in the pH range from 4.0 to 8.0, i.e., very similar to the PAC1 behavior. The pH dependence slope for the PAC3 polymeric form in the whole pH range was about 34 mV/pH, involving the transfer of a single hydrogen ion and two electrons (Table 1).

The limiting step of the redox reaction could be assessed according to the slope of bi-logarithmic *I_p_*–ν dependency. For the PAC1 monomer oxidation, it was 0.61, indicating quasi-diffusional control, whereas monomer reduction showed the slope of 0.71 (mixed adsorption–diffusion control of the reaction). At low scan rates, the PAC1 polymer redox signal demonstrated the slopes from 0.76 to 0.84 which also corresponded to the mixed adsorption–diffusion limitation of the electrode reaction. At higher scan rates, appropriate slopes were reduced by half to 0.41–0.42, corresponding to a bigger contribution of diffusion. At low scan rates, the oxidation and reduction slopes of the PAC2 polymeric form were close to 1 (1.11 and 1.07, respectively), concerning the surface confined limiting step of the electrode reaction. At higher scan rates, the PAC2 polymeric form slopes also declined to 0.77–0.79 (mixed adsorption–diffusion control). At low scan rates, the oxidation and reduction slopes of the PAC3 polymeric form were near 1 (0.89 and 0.97, respectively) demonstrating the prevalence of the adsorption influence. At higher scan rates, the PAC3 polymeric form redox signals were governed by diffusion (the slopes were 0.64–0.67, Table 1).

The Laviron Equations (1) and (2) [46] were used for the estimation of the electron transfer coefficients α and 1 − α. In the case of PAC2 and PAC3, they were near 0.5 for polymer oxidation, indicating the symmetrical transient state of the electron transfer equilibrium. For the PAC1 monomer oxidation and the PAC3 polymer reduction, electron transfer coefficients indicated the shift of the equilibrium of electron transfer to a more stable oxidation product. In the remaining cases, the α value exceeded 1.0, owing to a multi-electron or multistep electrode reaction for poly(Azure C) coatings [47] (Table 1).

(1) $$E_{pc}=E_{}^{0’}+\frac{RT}{\alpha nF}\ln\frac{RTk_{et}}{\alpha nF}-\frac{RT}{\alpha nF}\ln\nu$$

(2) $$E_{pa}=E_{}^{0\prime }+\frac{RT}{(1-\alpha )nF}\ln\frac{(1-\alpha )nF}{RTk_{et}}+\frac{RT}{(1-\alpha )nF}\ln\nu$$

*E_pa_* and *E_pc_* are here anodic and cathodic peak potentials; *E*^0′^ is the standard redox potential; *R* is the universal gas constant; *T* is the absolute temperature; *k_et_* is the heterogeneous constant of the electron transfer; *F* is the Faraday constant; *n* is the number of electrons transferred.

### 3.4. Scanning Electron Microscopy Measurements of PAC1 and PAC2 Coatings

**Figure 5:**
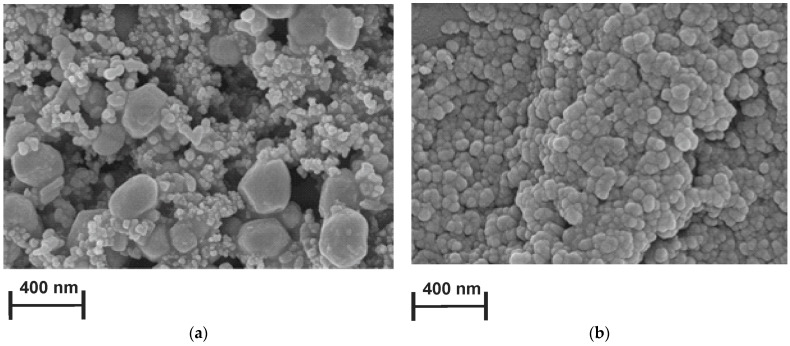
SEM images of the (**a**) SPCE covered with PAC2 and the (**b**) SPCE covered with PAC1.

SEM was used to estimate the morphological characteristics of bare and modified with PAC1 and PAC2 SPCEs. The surface of the bare electrode indicated distinctly visible carbon nanoparticles of about 40 nm in diameter (Figure S3). Morphology of the PAC coatings electrodeposited from reline and PB were strictly different from each other. In both cases, the carbon support was fully covered with polymer. Slower monomer diffusion in reline resulted in seeding fewer crystallization centers and hence in the formation of larger hexagonal particles surrounded partially with small glued particles (Figure 5a). In the aqueous medium, no diffusion hampering took place on the electropolymerization step, so the polymer coating formed a microgranular dense layer (Figure 5b). The difference shows new possibilities of tuning the morphology of the surface layer by performing electropolymerization in DES.

### 3.5. DNA Implementation Effect

**Figure 6:**
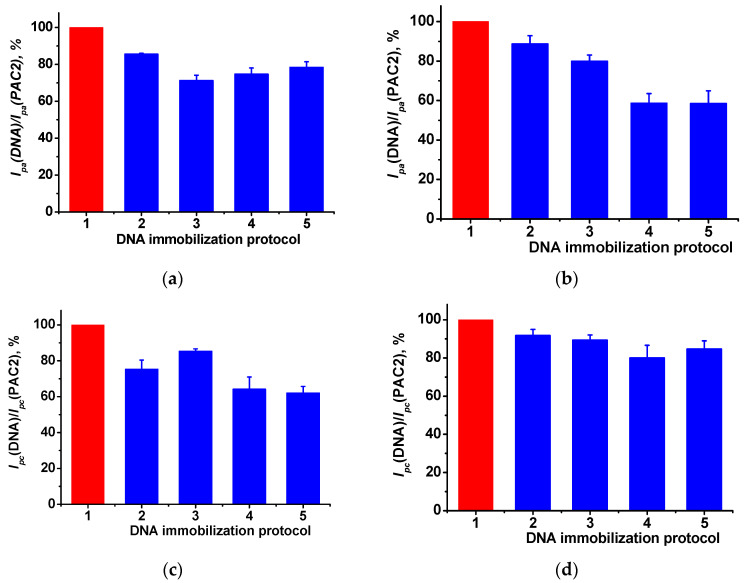
The relative changes of the peak currents of PAC2 and PAC2/DNA for (**a**) monomer and (**b**) polymer oxidation peaks, and (**c**) monomer and (**d**) polymer reduction peaks. DNA immobilization protocols: 1—PAC2 with no DNA, 2—DNA drying, 3, 4, 5—DNA incubation for 10, 20 and 30 min, respectively. Cyclic voltammetry, 0.1 M PB, pH 7.0, scan rate 0.15 V/s.

Double-stranded DNA from calf thymus was physically adsorbed on the PAC2 layer. For this purpose, 2 µL drop of 1 mg/mL DNA solution was incubated for 10–30 min or drop-casted on the electrode surface and dried. All the procedures were performed at ambient temperature. DNA implementation in the modifying layer resulted in notable changes in monomer and polymer peak currents (Figure 6a–d). Such an alteration was caused by polyelectrolyte complex formation between the positively charged PAC2 and negatively charged non-conductive double-stranded DNA molecules. Surface charge neutralization and DNA immobilization decreased the PAC2 peak currents recorded because of the electron transfer complication and the shift of the redox equilibrium on the electrode interface. Thus, the electrostatic control remarkably affects the voltammograms and can be considered as indirect evidence of the DNA implementation in the modifying layer.

### 3.6. Voltammetric Determination of Valrubicin

Interaction of valrubicin with double-stranded DNA interaction occurs via intercalation mechanism [21,33]. Valrubicin affects nucleic acids’ metabolism after entering cancer cells, causing cell cycle arrest in G2, inhibition of nucleosides incorporation in nucleic acids and interference of normal DNA topoisomerase-II activity [19].

In comparison with other anthracycline drugs, DNA–valrubicin interactions are weaker [19]. The binding constant *K_b_* of valrubicin to DNA depends on the DNA source. Thus, for fish sperm DNA, the *K_b_* estimated by spectrophotometric titration was equal to 1.75 × 10^3^ M^−1^ [21], for salmon testes DNA, to 1.81 × 10^5^, and for calf thymus DNA, to 4.99 × 10^4^ M^−1^ [25]. The valrubicin–DNA interaction changes the DNA strands volume and charge distribution in the electrode surface layer and hence can be detected by voltammetry.

**Figure 7:**
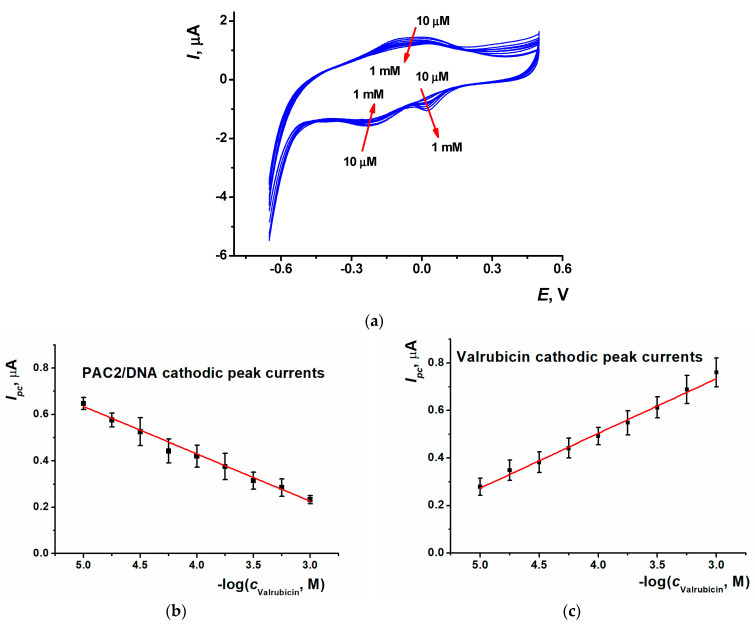
(**a**) Cyclic voltammograms recorded on the SPCE/PAC2/DNA after 20 min incubation in 10 µM, 18 µM, 30 µM, 56 µM, 0.1 mM, 0.18 mM, 0.3 mM, 0.56 mM and 1 mM valrubicin solution; 0.1 M PB, pH 7.0, scan rate 0.15 V/s. Arrows show the direction of the changes observed with increasing valrubicin concentration; calibration curves of valrubicin based on (**b**) the CV reduction peak current of PAC2/DNA coating and (**c**) the CV reduction peak current of valrubicin. Average ± S.D. values were calculated for twelve individual DNA sensors.

The SPCE/PAC2/DNA sensor was utilized for the quantification of valrubicin. 5 µL of valrubicin solution, of which the concentration ranged from 10 µM to 1 mM, were drop-casted on the modified electrode surface and left for 20 min, capped with plastic tubing to prevent drying. All of the procedures were performed at ambient temperature. Then, the drop was washed out and cyclic voltammogram was recorded in the range from −0.65 and 0.50 V at the scan rate of 0.15 V/s. For the valrubicin concentrations higher than 10 µM, two cathodic peaks appeared on the voltammogram, i.e., the reduction peak current of the PAC2/DNA coating (−0.23 V), which decreased against the DNA sensor prior to the contact with the drug, and the reduction peak current of valrubicin (+0.02 V), which increased with valrubicin concentration (Figure 7a). Synchronous changes of the peaks offer more reliable detection of specific DNA–valrubicin interactions, as was shown for other similar systems known as ratiometric sensors [48].

For the PAC2/DNA reduction peak current, the following calibration equation was obtained (Equation (3)). The LOD value was evaluated with the S/N = 3 criterion as 10 µM (Figure 7b,c).

*I_pc_*, µA = (1.4 ± 0.03) + (0.234 ± 0.008)log(*c*, M), R^2^ = 0.992 (3)

The calibration equation obtained for the valrubicin current is presented in Equation (4).

*I_pc_*, µA = (−0.38 ± 0.03) − (0.201 ± 0.008)log(*c*, M), R^2^ = 0.986 (4)

**Figure 8:**
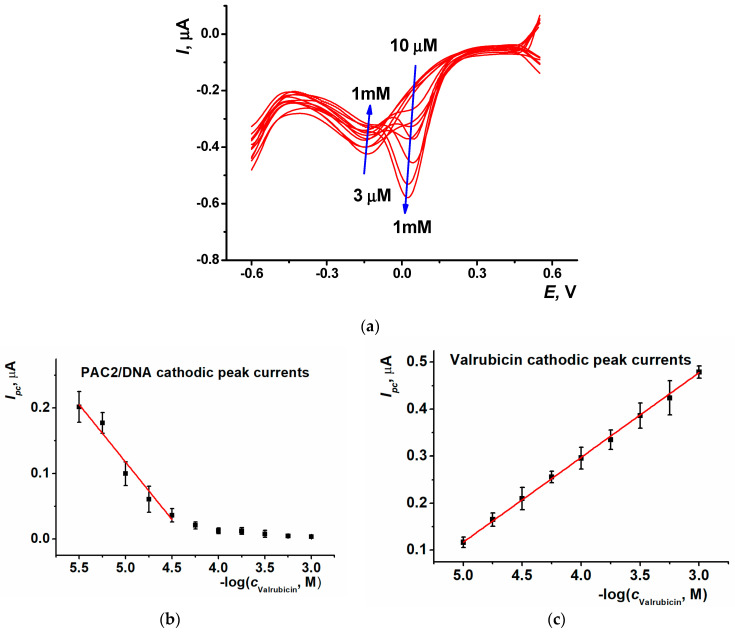
(**a**) SWV voltammograms recorded on the SPCE/PAC2/DNA after 20 min incubation in 3 µM, 5.6 µM, 10 µM, 18 µM, 30 µM, 56 µM, 0.1 mM, 0.18 mM, 0.3 mM, 0.56 mM and 1 mM valrubicin solution; 0.1 M PB, pH 7.0. Arrows show the direction of the changes observed with increasing valrubicin concentration; calibration curves of valrubicin based on (**b**) SWV reduction peak current of PAC2/DNA coating and (**c**) SWV reduction peak current of valrubicin. Average ± S.D. values were calculated for eight individual DNA-sensors.

To improve the sensitivity of valrubicin detection, SWV was used. Only the cathodic branch of the SWV voltammogram was recorded in the following conditions: 0.1 M PB, pH 7.0, potential scanning range from 0.55 to −0.60 V, accumulation time 5 s at 0.55 V, potential step 0.005 V, amplitude 0.002 V and frequency 20 Hz. Typical curves of valrubicin detection in its concentration range 3 µM–1 mM are shown in Figure 8a and calibration curves in Figure 8b,c.

It should be noted that the valrubicin peak currents increased with the drug concentration both in the CV and SWV mode. Contrary to that, the signals of the PAC2/DNA coating showed the opposite direction of the change. Higher valrubicin concentrations corresponded to smaller peak currents and partial overlapping with the valrubicin peaks interfered with the signal measurement after reaching 30 μM. For this reason, the linear range reached in SWV mode was narrower than that in CV mode.

The calibration equation of valrubicin determined by the reduction peak currents of the PAC2/DNA coating and by own reduction peaks of the drug are presented in Equations (5) and (6), respectively. The LOD assessed for S/N = 3 was equal to 1 µM.

*I_pc_*, µA = (0.78 ± 0.05) − (0.179 ± 0.020)log(*c*, M), R^2^ = 0.952 (5)

*I_pc_,* µA = (1.005 ± 0.05) + (0.177 ± 0.002)log(*c*, M), R^2^ = 0.999 (6)

Although the use of PAC coatings did not allow us to reach the sensitivity of valrubicin determination shown by its direct oxidation on the electrodes covered with nanomaterials [24,25], the DNA sensor developed has some advantages over existing analogs, i.e., the direct modeling of target DNA–drug interactions that takes into account the accessibility of the biopolymer for small molecules intercalation, easy assembling with minimal number of steps and reactant consumption, and a very small volume of the sample required for assay that is performed in one drop on the SPCE interface. In addition, it should be mentioned that cathodic reduction excludes interference with oxidizable components of biological fluids which are considered as a main drawback of conventional electrochemical sensors for small molecules detection. Low working potential prolongs the lifetime of the sensor because of a higher stability of the modifier and excludes deterioration of the polymer film due to the internal redox reaction initiated during the measurement. The ratiometric protocol of the signal assessment improves the durability of the sensor via synchronous monitoring of the signal changes.

The analysis of urine after the administration of 14 doses of valrubicin (800 mg) showed 99% recovery within 24 h, yielding an average concentration of 0.2 mM [17]. Metabolites cover only 0.4% of the dose. In serum, valrubicin is metabolized within 2 h of retention. Thus, the concentrations detected with the DNA sensors developed are sufficient for the reliable control of the valrubicin treatment in bladder cancer treatment.

### 3.7. Measurement Precision and Real Sample Analysis

Sensor-to-sensor repeatability was assessed using a set of eight SPCEs modified with the same reagents. The relative standard deviation (R.S.D.) of the peak currents recorded was found to be 4.4% (0.1 mM valrubicin) and the period of the 25% decrease in the signal was 20 days when stored in dry conditions at 4 °C. The electrochemical activity of the PAC layer with no DNA in the surface layer was observed for at least 90 days. The R.S.D. of the signal of valrubicin detection increased twice at the end of the storage period.

To estimate the usability of the SPCE/PAC2/DNA sensor, several artificial and real biological samples spiked with valrubicin were tested. Preliminary, some common compounds present in the biological fluids were tested in the model solution and no influence of 0.1 mM glucose, urea and lactate was found on the PAC2 redox signals and valrubicin reduction. Then, for the artificial and human urine samples, Ringer-Locke solution and human saliva were considered. The SWV determination of valrubicin was performed for two medication concentrations (10 μM and 0.1 mM), corresponding to various scenarios of drug administration and corresponding to different linear ranges of appropriate calibration dependencies plotted for PAC and own valrubicin reduction peaks (Table 2). For artificial urine, Ringer-Locke solution and saliva samples were analyzed without additional treatment or dilution. The protocol took only 10 min for incubation. Human urine should be diluted in 1:1 ratio with a PB buffer to eliminate the influence of organic and inorganic urine components [49].

Satisfactory recovery in the range from 90 to 109% was established for the samples tested. The use of the PAC cathodic peak led to some higher deviation of the recovery against valrubicin reduction. In the case of human saliva, deviation reached 45%, which is unacceptable for valrubicin determination goals. It could be attributed to the complex compound of saliva which selectively affected the polymer coating signal [50,51]. Meanwhile saliva is rather rarely used in the monitoring of the drug intravesical administration.

## 4. Conclusions

In this work, a voltammetric DNA sensor based on PAC electropolymerized from reline on SPCE has been developed for valrubicin determination. This is the first work considering the use of DES as a medium for electropolymerization of Azure C which offered new opportunities for the determination of valrubicin as a DNA intercalator. This approach allowed us to avoid the use of organic solvents for monomer dissolution and to decrease the volume of reaction media to 100 μL. Together with the use of disposable SPCEs, this made it possible to significantly decrease possible contamination of the environment with toxic species.

The use of DES as electropolymerization media made it possible to intensify the polymerization product due to the increased concentration of the monomer. The film obtained showed higher granulation and the ability to accumulate DNA by electrostatic adsorption. The comparison of electron transfer parameters confirmed the reversible character of the surface redox process and its sensitivity to the DNA influencing factors. Valrubicin as an intercalating agent changed the size and charge distribution of the DNA molecule and hence shifted the redox equilibrium on the electrode surface. This resulted in remarkable changes in the peak currents attributed to the polymeric layer. In addition, own peaks of valrubicin reduction appeared on voltammograms so that synchronous changes of the currents improved the reliability of the signal.

The DNA sensor developed was successfully applied to the determination of the valrubicin concentration in the range from 3 µM to 1.0 mM (LOD 1 µM). All of the measurements were performed at ambient temperature. The sensitivity achieved was sufficient for the monitoring of the drug release in urine, as was shown by testing on the spiked artificial and real urine samples. No influence of common components of biological fluids was found and the recovery of 90–110% was established for all of the samples tested. Simple assuming, green chemistry approach with no toxic reagents and organic solvents, and reliable detection from small volumes of biological samples can be mentioned as advantages of the DNA sensor proposed. After testing on clinical samples, it might find further application in pharmacokinetics studies, new anticancer drug screening and personal doses of drug selection for the chemotherapy of cancer.

## Figures and Tables

**Table 1:** Electrochemical characteristics for the PAC1, PAC2 and PAC3 coating. Linear range of parameters is given in the brackets.

Coating Parameter	Peak on CV	PAC1	PAC2	PAC3
d(*E_m_*, V)/dpH	Monomeric form	−0.085 ± 0.005 (pH = 2.0–4.0) −0.047 ± 0.002 (pH = 4.0–8.0)		−0.080 ± 0.000 (pH = 2.0–4.0) −0.042 ± 0.002 (pH = 4.0–8.0)
	Polymeric form	−0.064 ± 0.003 (pH = 2.0–5.0) −0.024 ± 0.003 (pH = 5.0–8.0)	−0.039 ± 0.000 (pH = 2.0–7.0) −0.054 ± 0.004 (pH = 7.0–10.0)	−0.034 ± 0.001 (pH = 2.0–8.0)
d(log*I_p_*)/d(logν)	Monomer oxidation	0.63 ± 0.01		
	Monomer reduction	0.71 ± 0.02		
	Polymer oxidation	0.76 ± 0.04 (0.01–0.1 V/s) 0.41 ± 0.04 (0.1–0.5 V/s)	1.11 ± 0.02 (0.01–0.08 V/s) 0.77 ± 0.01 (0.08–0.5 V/s)	0.89 ± 0.07 (0.01–0.1 V/s) 0.64 ± 0.06 (0.1–0.5 V/s)
	Polymer reduction	0.84 ± 0.01 (0.01–0.1 V/s) 0.42 ± 0.03 (0.1–0.5 V/s)	1.07 ± 0.05 (0.01–0.08 V/s) 0.79 ± 0.03 (0.08–0.50 V/s)	0.97 ± 0.01 (0.01–0.1 V/s) 0.67 ± 0.02 (0.1–0.5 V/s)
d*E_p_*/d(logν)	Monomer oxidation	−0.073 ± 0.016		
	Polymer oxidation	0.030 ± 0.003	0.094 ± 0.003	0.104 ± 0.005
	Polymer reduction	−0.043 ± 0.001	−0.035 ± 0.011	−0.068 ± 0.003
Electron transfer coefficient	Monomer oxidation	α = 0.81		
	Polymer oxidation	(1 − α) = 1.97	(1 − α) =0.63	(1 − α) = 0.57
	Polymer reduction	α = 1.37	α = 1.70	α = 0.87

**Table 2:** Valrubicin determination in spiked samples mimicking blood plasma, artificial urine and in real human urine, and saliva samples by SWV reduction peak current (*I_p_*) changes of PAC2/DNA. Average ± S.D. for six individual sensors.

Sample	*I_p_*, µA	Recovery, %
10 µM valrubicin (standard solution: *I_pc_* = 0.100 ± 0.028 µA)		
Artificial urine	0.093 ± 0.011	93 ± 11
Ringer-Locke solution	0.082 ± 0.020	82 ± 20
Human saliva	0.110 ± 0.045	110 ± 45
Human urine (1:2 dilution)	0.083 ± 0.016	83 ± 6
0.1 mM valrubicin (standard solution *I_pc_* = 0.296 ± 0.030 µA)		
Artificial urine	0.297 ± 0.045	100 ± 15
Ringer-Locke solution	0.267 ± 0.033	90 ± 11
Human saliva	0.303 ± 0.032	102 ± 11
Human urine (1:2 dilution)	0.321 ± 0.051	109 ± 17

## Data Availability

The data presented in this study are available in supplementary materials.

## Supplementary Materials

The following supporting information can be downloaded at: https://www.mdpi.com/article/10.3390/bios13100931/s1, Figure S1: Changes in cyclic voltammograms of PAC1–PAC3 under the signal stabilization, Figure S2: The pH dependence of the peak currents attributed to the monomeric and polymeric forms of Azure C in the layer; Figure S3: SEM image of the surface of the working electrode.
