# Supplementary material for: Electrochemical DNA Sensor for Valrubicin Detection Based on Poly(Azure C) Films Deposited from Deep Eutectic Solvent
Source: Biosensors (Basel). 2023 Oct 18;13(10):931. doi: 10.3390/bios13100931 (PMC10605098; doi:10.3390/bios13100931)
Supplement: Supplementary file 1 [file biosensors-13-00931-s001.zip › biosensors-2613956-supplementary.pdf]

Electronic Supporting Information

To the article of Porfireva A.V., Begisheva E.A., Evtugyn V.G., Evtugyn G.A. "Electrochemical DNA-Sensor for Valrubicin Detection Based on Poly(Azure C) Films Deposited from Deep Eutectic Solvent"

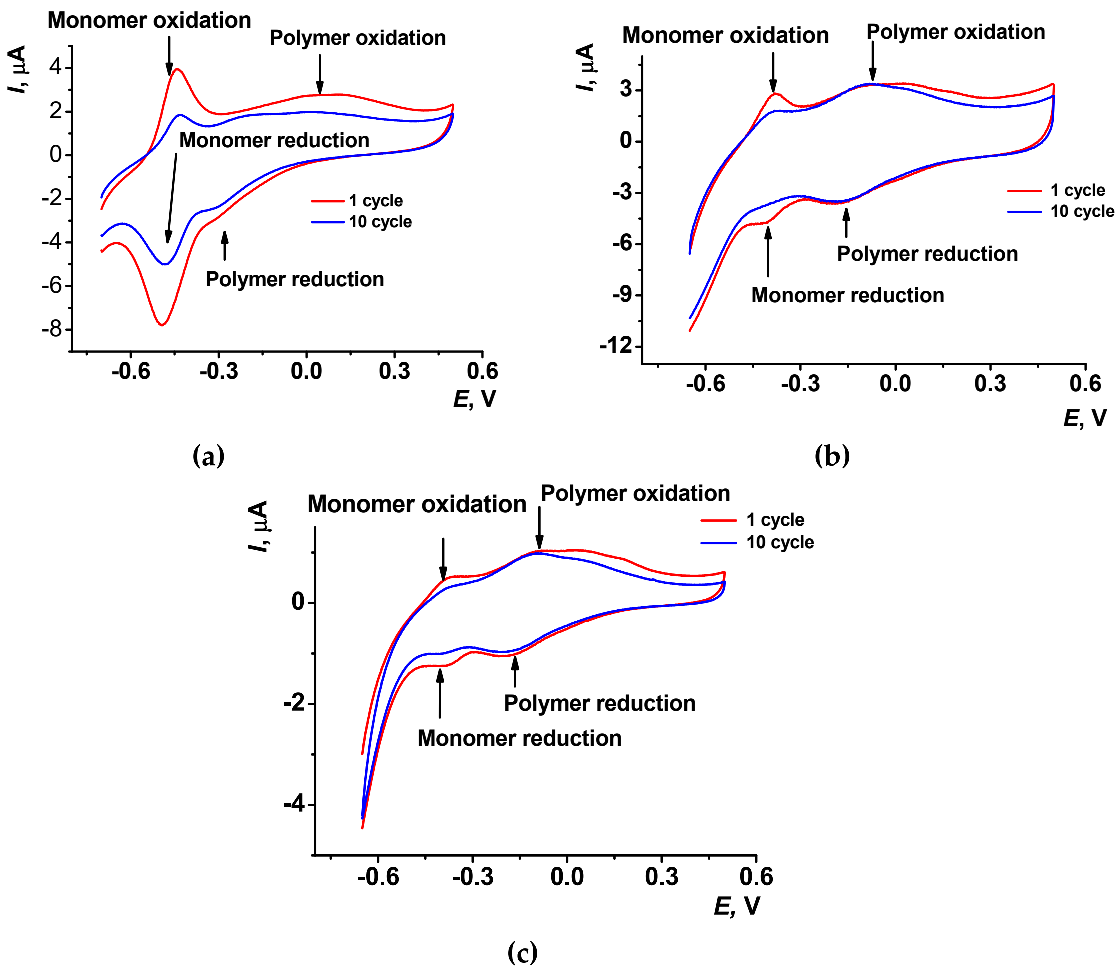

Figure S1. Changes in cyclic voltammograms of (**a**) PAC1, (**b**) PAC2, (**c**) PAC3 layers under the signal stabilization. First and tenth voltammograms are shown. 0.1 M PB, pH = 7.0, between −0.7 and 0.5 V for PAC1, between −0.65 and 0.5 V for PAC2 and PAC3, 0.15 V/s.

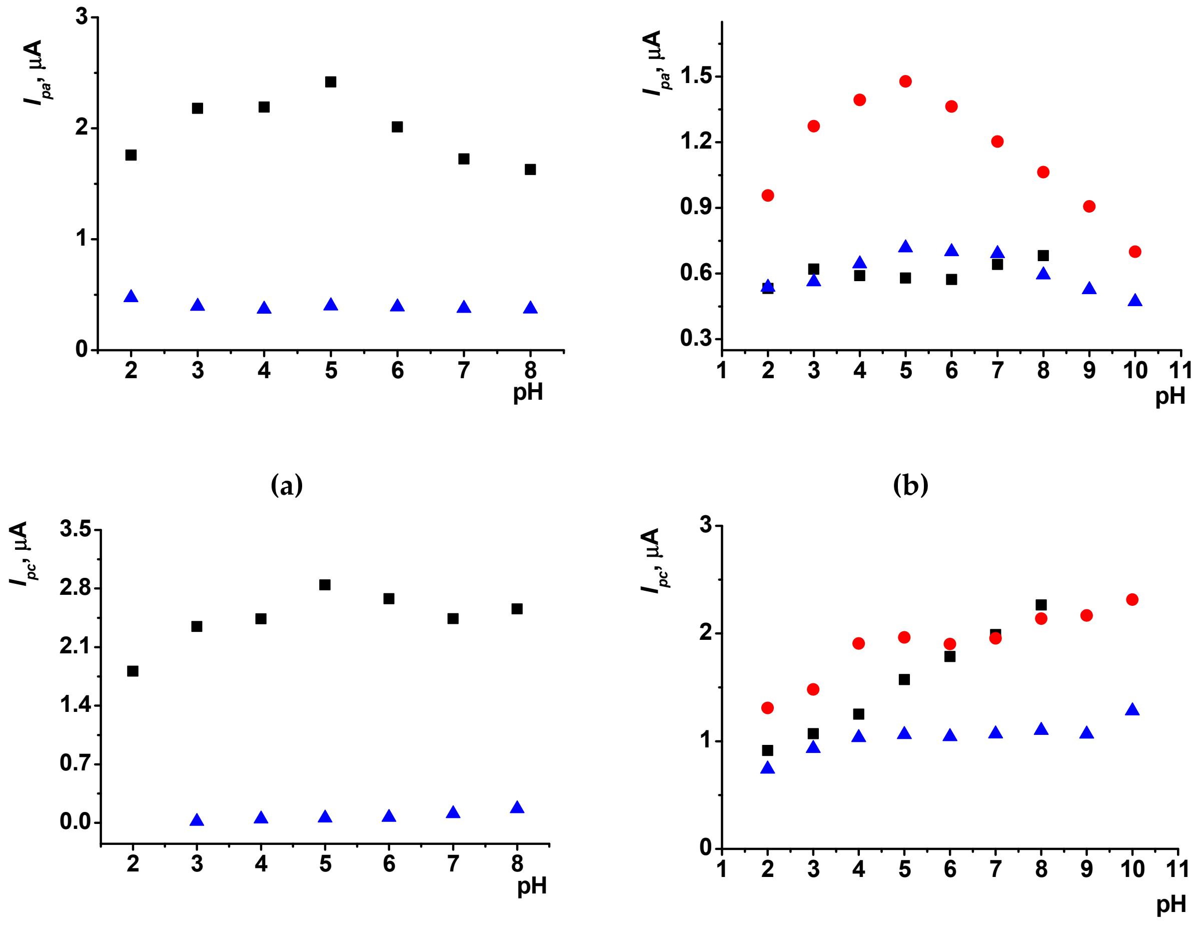

(a) (b)

(c) (c)

Figure S2. The pH dependence of the monomer (**a**) and polymer (**b**) oxidation peak currents, monomer (**c**) and polymer (**d**) reduction peak currents. Measurements in 0.1 M PB, pH 2.0–10.0. SPCE modified with PAC1 (black symbols), PAC2(red symbols) and PAC3 (blue symbols).

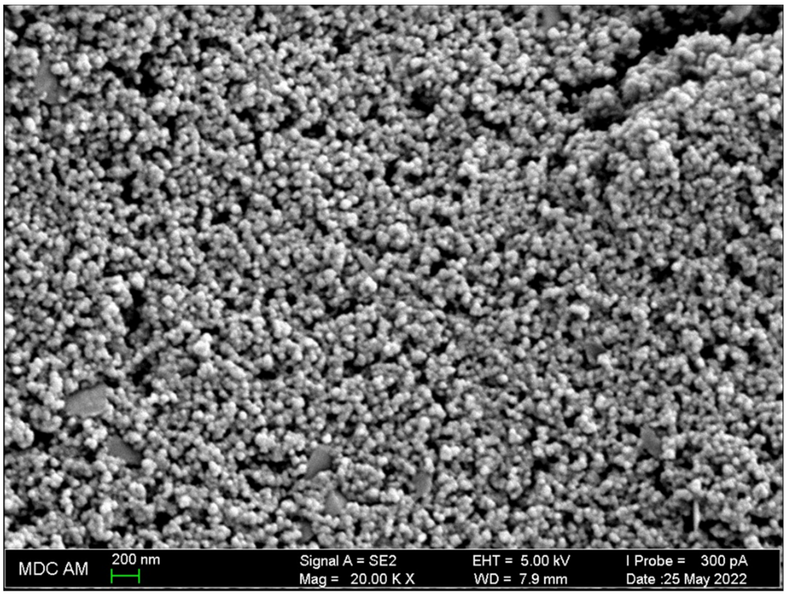

Figure S3. SEM image of the bare SPCE
